# Peroperative epicardial ultrasonography of distal coronary artery bypass graft anastomoses using a stabilizing device. A feasibility study

**DOI:** 10.1186/s13019-020-1057-x

**Published:** 2020-01-08

**Authors:** Jan Jesper Andreasen, Dorte Nøhr, Alex Skovsbo Jørgensen, Poul Erik Haahr

**Affiliations:** 1 Department of Cardiothoracic Surgery, Aalborg University Hospital, Hobrovej 18-22, 9000 Aalborg, Denmark; 2 Clinical Institute, Aalborg University, Sdr. Skovvej 15, 9000 Aalborg, Denmark; 3 Department of Health Science and Technology, Aalborg University, Fredrik Bajers Vej 7, 9220 Aalborg, Denmark

**Keywords:** Epicardial ultrasonography, Coronary bypass surgery, Coronary anastomosis, Quality assessment

## Abstract

**Background:**

Widespread use of intraoperative epicardial ultrasonography (ECUS) for quality assessment of coronary artery bypass graft anastomoses during coronary artery bypass grafting (CABG) has not occurred - presumably due to technological and practical challenges including the need to maintain stable and optimal acoustic contact between the ultrasound probe and the target without the risk of distorting the anastomosis. We investigated the feasibility of using a stabilizing device during ultrasound imaging of distal coronary bypass graft anastomoses in patients undergoing on-pump CABG. Imaging was performed in both the longitudinal and transverse planes.

**Methods:**

Single-centre, observational prospective feasibility study among 51 patients undergoing elective, isolated on-pump CABG. Ultrasonography of peripheral coronary bypass anastomoses was performed using a stabilizing device upon which the ultrasound transducer was connected. Transit-time flow measurement (TTFM) was also performed. Descriptive statistical tests were used.

**Results:**

Longitudinal and transverse images from the heel, middle and toe were obtained from 134 of 155 coronary anastomoses (86.5%). After the learning curve (15 patients), all six projections were obtained from 100 of 108 anastomoses scanned (93%). Failure to obtain images were typical due to a sequential curved graft with anastomoses that could not be contained in the straight cavity of the stabilizing device, echo artefacts from a Titanium clip located in the roof of the anastomoses, and challenges in interpreting the images during the learning curve. No complications were associated with the ECUS procedure. The combined ECUS and TTFM resulted in immediate revision of five peripheral anastomoses.

**Conclusions:**

Peroperative use of a stabilizing device during ultrasonography of coronary artery bypass anastomoses in on-pump surgery facilitates imaging and provides surgeons with non-deformed longitudinal and transverse images of all parts of the anastomoses in all coronary territories. Peroperative ECUS in addition to flow measurements has the potential to increase the likelihood of detecting technical errors in constructed anastomoses.

**Trial registration:**

The study was registered on September 29, 2016, ClinicalTrials.gov ID: NCT02919124.

## Background

Intraoperative quality assessment of peripheral coronary anastomoses during coronary artery bypass grafting (CABG) offers surgeons the opportunity to detect technical failures mandating revision of the bypass graft before chest closure. Several methods for quality assessment are in use, including intraoperative selective coronary angiography, transit-time flow measurement (TTFM), intraoperative fluorescence imaging and high-frequency epicardial ultrasonography (ECUS) [1–3].

High-frequency intraoperative ECUS has been used for more than three decades [4], and this technique has shown to be complementary to TTFM for improving the diagnostic accuracy during graft assessment [5]. However, widespread use of intraoperative ECUS for quality assessment of coronary artery bypass graft anastomoses during CAGB procedures has not been adopted, presumably due to technological and practical challenges.

Among these challenges are maintaining stable and optimal acoustic contact between the ultrasound probe and examining the anastomosis without the risk of distorting it. To overcome these challenges, we developed an ultrasound transducer positioning device. This device can be used for stabilizing the involved part of the myocardium on the contracting/beating heart and for positioning of the ultrasound transducer correctly for imaging of anastomoses in the longitudinal and transverse planes without deforming the anastomoses [6].

The primary objective of the present study was to investigate the ultrasound imaging procedure’s feasibility in producing images of coronary artery bypass anastomoses in patients undergoing on-pump CABG both in the longitudinal and transverse planes using a stabilizing device. The secondary aims were to evaluate the safety of the procedure and to register whether surgeons revised any of the coronary anastomoses based on the findings from ECUS and TTFM.

We hypothesized that use of the stabilizing device would safely facilitate ECUS imaging of the heel, middle and toe of at least 80% of all peripheral coronary bypass anastomoses both in the longitudinal and transverse planes during on-pump CABG.

## Methods

### Patients

This was a single-centre, observational prospective feasibility study among patients undergoing elective, isolated on-pump CABG in the Department of Cardiothoracic Surgery, Aalborg University Hospital, Denmark. Between 100 and 150 isolated, elective CABG procedures (approximately 20% off-pump) are performed each year. The study was approved by the Regional Ethical Committee (N-20160012), the Danish Medicines Agency (ref. 2,016,020,479), and the Danish Data Protection Agency (2008-58-0028). Written informed consent was obtained from all participants. Trial registration: ClinicalTrials.gov ID: NCT02919124; Registered September 29, 2016.)

Inclusion criteria were elective patients with an age ≥ 18 years scheduled for on-pump CABG. Logistic euroSCORE II should be < 6, and surgery was to be performed by a non-trainee surgeon in order to keep the time for surgery including the cross-clamp and perfusion time as low as possible during the study.

Exclusion criteria were conversion to or planned off-pump surgery and urgent or emergent surgery. ECUS was cancelled and the patient excluded from the study if the surgeon decided to end the surgery as fast as possible due to haemodynamic challenges or peroperative complications. Other exclusion criteria were pregnant or lactating patients.

Clinical characteristics and preoperative laboratory data were collected in all patients: age, gender, height and weight, p-creatinine, estimated glomerular filtration rate, hypertension requiring medical treatment, diabetes, hypercholesterolemia requiring treatment, history of atrial fibrillation, smoking status (active, stopped, or never smoked), left ventricular ejection fraction, logistic euroSCORE II, number of diseased (≤50% stenotic) coronary vessels (1–2 or 3 vessel disease), > 50% left main stenosis, and preoperative antithrombotic or anticoagulation medication within 5 days.

Fractional flow reserve measurement was performed if there was a suspicion of a coronary artery stenosis being non-significant. In general, patients continued ongoing β-blocker and acetylsalicylic acid treatment until surgery, but other antithrombotic drugs and orally anticoagulants were discontinued before surgery.

### Surgical procedure and postoperative medical treatment

Surgery was performed on-pump, and routine surgical, anaesthetic and cardiopulmonary bypass procedures were employed. All patients received perioperative prophylactic anti-fibrinolytic treatment with 1.5 g tranexamic acid (Stragen Nordic A/S) as an intravenous bolus followed by a constant infusion of 200 mg/h until 1.5 g. In all cases, transoesophageal echocardiography was available. Cold blood cardioplegia was administered in the aortic root every 20 min during aortic cross-clamping. Additional cardioplegia was infused directly into a free graft just after an anastomosis was performed, keeping the flow approximately 60 ml/min and the pressure below 100 mmHg. The graft compositions and types of conduits used were at the discretion of the surgeon. In general, the left internal mammary artery (LIMA) was anastomosed to the left anterior descending artery (LAD) whenever this vessel was to be grafted. LIMA and the right internal mammary artery (RIMA) were harvested either skeletonised or non-skeletonised and used as either in situ grafts or free grafts. Both single and sequential grafts were used. Radial arteries (RAs) were harvested using an open technique and in general used on the left-sided coronary arteries if the target vessel was at least 70% stenotic. Saphenous vein grafts (SVGs) were harvested using either an open or endoscopic technique. Side branches from all conduits were closed using titanium ligation clips (Symmetry Surgical, Germany). All RAs and SVGs were gently flushed after harvesting and kept at room temperature in a blood-based solution containing saline, heparin (SAD, Amgros I/S, Denmark) and papaverin (Aalborg University Hospital, Denmark).

A decision to revise a peripheral anastomosis was based on the combined findings from TTFM and ECUS and left to the discretion of the surgeon, who also considered the quality of the target vessel.

If an anastomosis was to be revised, the graft was divided just proximal to the heel of the anastomosis. The roof of the anastomosis was opened to inspect the inner part of the anastomosis and search for a possible technical error inside the anastomosis.

The following surgical data were collected: date of surgery, duration of surgery (skin-to-skin), cross-clamp and perfusion time, number and types of peripheral anastomoses (end-to-end or side-to-side), target vessels, graft materials and graft compositions used.

Patients receiving a RA graft were treated with a continuous intravenous administration of Nifedipine (Adalat® pro infusione, Bayer Pharma AG, Germany) on the day of surgery followed by tablets of Nifedipine (Adalat® Oros, Bayer A/S, Denmark), 30 mg, administered once daily for 6 months. All patients received tablets of acetylsalicylic acid (Takeda Pharma A/S, Denmark), 75 mg, once daily starting on the day of surgery. Patient with a recent preoperative myocardial infarction or unstable angina and patients who recently underwent percutaneous coronary intervention also received tablets of either Ticagrelor (AstraZenica A/S, Denmark), 90 mg b.i.d., or Clopidogrel, 75 mg once daily (Sandoz A/S, Denmark), for 12 months, starting when subcutaneous injections of Deltaparin (Pfizer ApS, Denmark) were discontinued.

### High-resolution epicardial ultrasonography using a stabilizing device

**Fig. 1:**
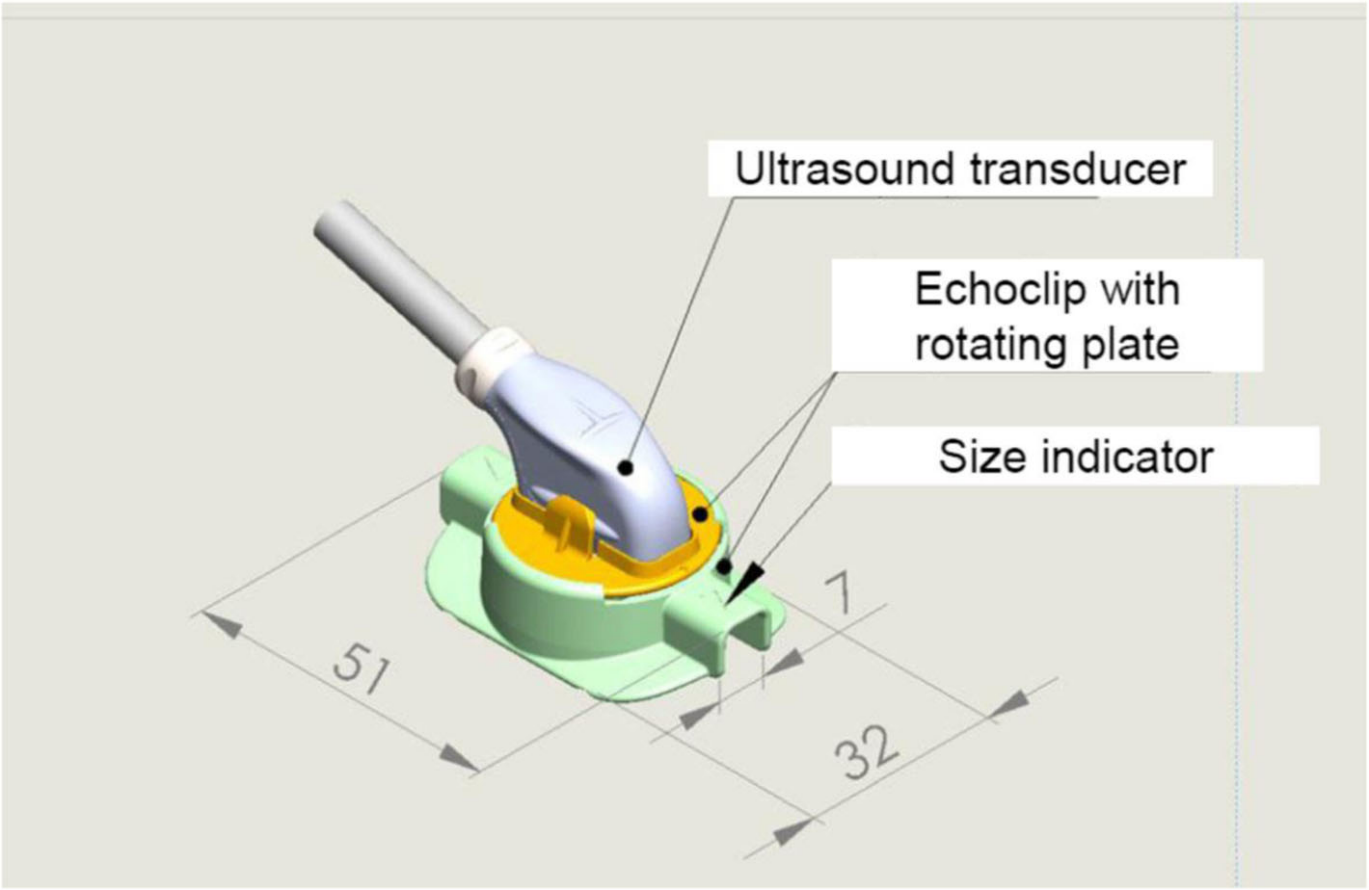
Schematic drawing of the Echoclip device (Numbers are in millimetres)

The Medistim VeriQ™ System with a 15-MHz ultrasonography probe was used (Medistim A/S, Oslo, Norway). The ultrasonography probe was connected to a rotatable plate of a stabilizing device (Echoclip, Aalborg University Hospital, Denmark) (Fig. 1). The stabilizing device was designed to receive sterile gel for ultrasonography in the cavity together with the conduit/anastomosis to be evaluated ensuring air-free contact between the transducer and the anastomosis of interest. Rotation of the plate to which the probe was attached allowed ultrasonography of an anastomosis in different planes. The device, which is not yet commercially available, is a disposable plastic article tested for biocompatibility according to international standard ISO 10993-1. The device was produced with different cavity sizes designed to receive conduits/anastomoses with different diameters, i.e., from 1 mm – 7 mm. This allows anastomoses to be imaged without applying pressure on the graft when stabilizing the heart around the anastomosis with the stabilizer. The device was created with the intention of allowing ultrasound gel in excess to escape from the cavity during scanning, reducing the risk of gel being squeezed into the anastomosis due to an increased pressure around the anastomosis.

**Fig. 2:**
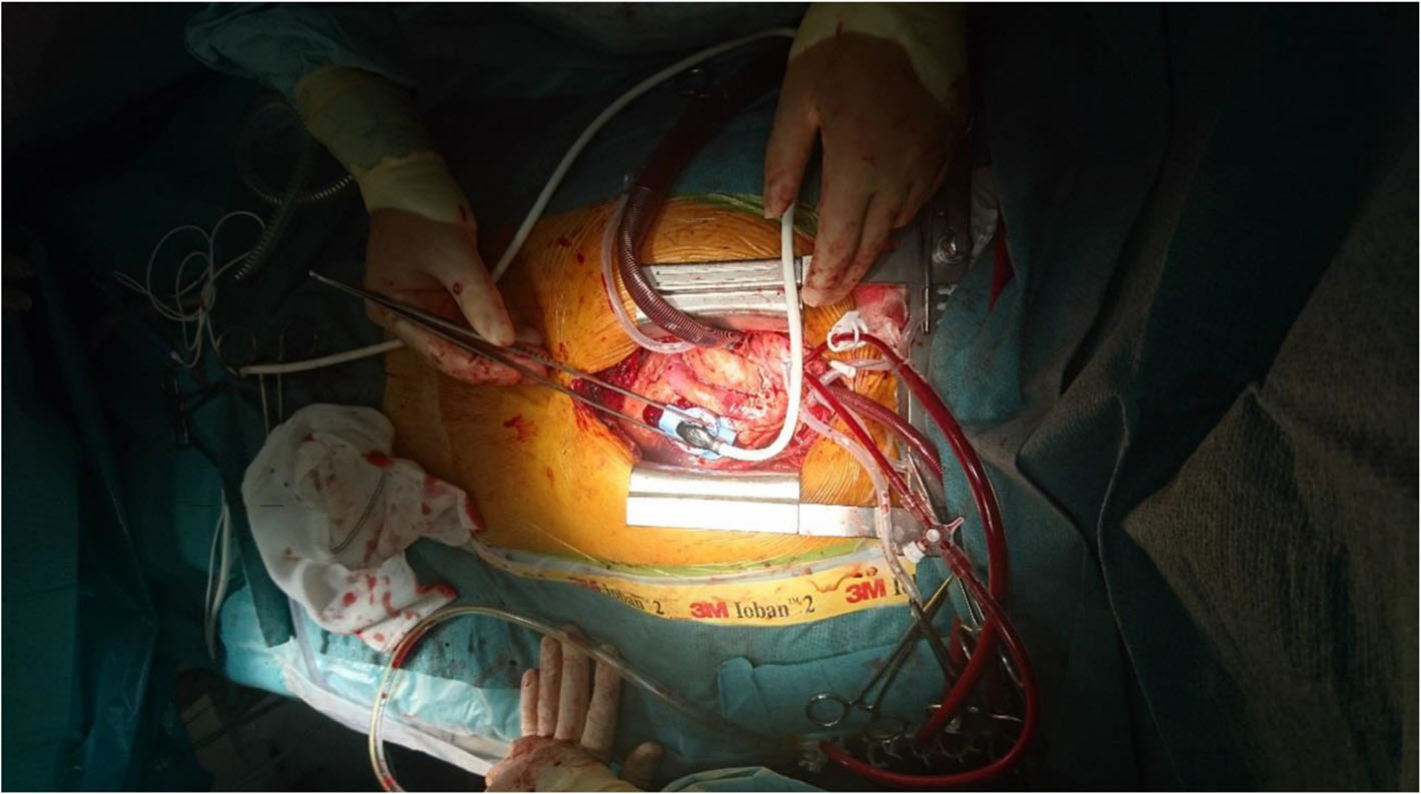
Epicardial ultrasonography of the left internal mammary artery anastomosis to the left anterior descending coronary artery in the longitudinal plane

All peripheral anastomoses except in situ internal artery mammary anastomoses were assessed during aortic cross-clamp while infusing cold blood cardioplegia directly into the free graft just after completion of an anastomosis. ECUS was performed in in situ IMA anastomoses after the cross-clamp was released while still on-pump (Fig. 2). We only searched for three specific anatomical parts of the anastomosis (heel, middle and toe) and did not aim for a precise evaluation to identify potential graft failures, as we were not experienced in interpretation of these ultrasonography images in relation to graft failure.

The surgeon would ensure that the graft was located in the cavity of the stabilizer in order to avoid obstruction of the graft flow during the scanning procedure. Once the stabilizing device with gel was located over the graft and anastomosis, we tried to identify longitudinal images of the heel, middle and toe of the anastomosis using both Doppler colour flow and 2D imaging. After capturing images in the longitudinal plane, the rotating disc with the transducer was then turned and aligned with the coronary artery grafted to obtain transverse images of the anastomoses. In this transverse plane, we again tried to identify the heel, middle and toe of the anastomosis. We considered use of the Echoclip device to be successful if we were able to obtain images in which the inner vessel wall of all three parts of an anastomosis (heel, middle and toe) could be identified in at least 80% of all peripheral anastomoses in both the longitudinal and transverse planes.

Instillagel®, Farco-Pharma, or the Safergel-sterile ultrasonic gel® Safersonic were used as ultrasound gel. However, after inclusion of the initial four patients we became aware that no ultrasound gel had been approved for application in the pericardium during cardiac surgery even though gel has been used for many years in the pericardium during both ECUS [7] and TTFM [8]. The study was therefore put on hold for several months as we were unable to identify any ultrasound gel approved for application in the pericardium. Later, we developed a technique during which we used coagulated blood obtained from the patient before heparinization instead of gel in order to obtain acoustic contact with the anastomoses [6]. In brief, 20–40 ml of blood was collected from the patient through a central venous catheter and from the surgical field before heparinization. The blood was left to clot in a sterile plastic cup and later in a metal cup as the blood did not coagulate sufficiently when stored in the plastic cup. Coagulated blood was sucked away from the pericardial space with a wall suction after the imaging procedure.

Initially, all intraoperative graft assessments using the Echoclip device were performed by the operating surgeon, but after 15 surgeries all intraoperative graft verifications by ECUS were performed by only one investigator in order to overcome the learning curve as fast as possible.

It was registered if the device resulted in or was be suspected to be involved in an accident or incident regardless of whether the cause was a device malfunction or misuse.

### Transit time flow measurements

TTFM was performed at different time points during surgery according to the discretion of the surgeon in addition to ECUS. The first time was after release of the cross-clamp when all proximal and distal anastomoses were performed. The second was after weaning from ECC before protamine administration, and the third was after protamine administration. The assessment of graft failure using TTFM was based on flow curves together with information regarding diastolic filling percentage (DF%), pulsatility index (PI), mean flow values and the blood pressure. No definitive cutoff values defining graft-failures were used, but in general the surgeons suspected a potential graft failure if the off-pump flow was < 20 ml/min and/or if the PI was > 5. These cut-off values have previously been suggested in the 2014 ESC/EACTS Guidelines on myocardial revascularization [9].

### Clinical outcome measures

The following in-hospital and 30-day outcomes were registered: perioperative myocardial infarction defined as an increase of a biomarker value (Creatinine kinase isoenzyme-MB) to above five times the 99th percentile of the normal reference range associated with either the appearance of a new pathological Q-wave, new left bundle branch block, angiographic documented graft, native coronary artery occlusion, or imaging evidence of new loss of viable myocardium [10]; development of acute kidney injury based on the Kidney Disease Improving Global Outcomes criteria, i.e., an increase in p-creatinine ≥26.5 μmol/l within 48 h or an increase ≥1.5 times baseline [11]; new onset atrial fibrillation identified by continuous electrocardiographic (ECG) monitoring or a 12-lead ECG; and graft failure identified by coronary angiography. We also registered the need for re-intervention due to bleeding or myocardial ischaemia, development of transitory cerebral ischaemia (TCI) or stroke confirmed by computed tomography or magnetic resonance scanning, and deep sternal wound infection was defined as positive culture results of the sternal wound or drainage from the mediastinal area. The in-hospital and 30-day mortality was also registered.

### Statistical analyses

A total of 100 patients was planned to be enrolled. The number of patients to be included was arbitrarily decided. No formal power calculation was performed as this was a descriptive feasibility study and the Echoclip device had never been used in patients before. With an estimated average of 2.5 peripheral anastomoses per patient and a possible drop-out of 10% of the patients, we expected to obtain images of approximately 225 peripheral anastomoses in 90 patients.

The proportion of successful anastomoses imaged is given as a percentage of the total number of peripheral anastomoses performed. Continuous values are expressed using the median and interquartile range and categorical variables as percentages. Descriptive statistical data were retrieved from a REDCap database (Research Electronic Data Capture; Vanderbilt University, USA).

## Results

A total of 56 patients were included in the study between September 27, 2016 – March 4, 2019. Five patients were excluded: two patients underwent off-pump surgery due to the surgeons’ preferences, one patient underwent percutaneous coronary intervention instead of CABG due to the patient’s condition on the day of surgery, one patient needed concomitant aortic valve surgery, and the log euroSCORE II was > 6 in one patient. Thus, 51 patients with 155 peripheral coronary anastomoses were available for analyses. The median age of the patients was 70 years (interquartile range: 60.5–73.0 years). We decided to stop recruiting patients after including 56 non-consecutive patients as we did not obtain any further technical or practical information on how to use the Echoclip device. A team of six surgeons performed all operations, but one attending surgeon performed the majority of the ECUS procedures. The 5-mm Echoclip device was most often used, and the 1-mm Echoclip device was never used. The learning curve for obtaining images from anastomoses in different graft compositions involved approximately 15 patients as imaging became easier, faster and more precise after approximately 15 patients.

Patient characteristics regarding patients in whom ECUS were performed are shown in Table 1. As expected, men were more prominent.

**Table 1:** Preoperative patient characteristics (*n* = 51)

Female/Male	9 (17.6%) / 42 (82.4%)
Age (years; Median, quartile)	70 (60.5–73.0)
Body mass index (Median, quartile)	27.4 (25.2–30.8)
Hypertension	42 (82.4%)
Diabetes	17 (33.3%)
History of atrial fibrillation	(6, 11.8%)
Smoke	
Never	11 (21.6%)
Active	11 (21.6%)
Previous	29 (56.8%)
Hypercholesterolemia	44 (86.3%)
Recent myocardial infarction (< 3 months)	8 (15.7%)
Left ventricular ejection fraction (Median, interquartile range)	60% (50–60%)
p-creatinine (μmol/l) (Median, interquartile range)	83 (73–94.5)
eGFR (ml/min)	80 (70–90)
Coronary disease (n and %)	
1 vessel	2 (3.9%)
2 vessels	13 (25.5%)
3 vessels	36 (70.6%)
Left main stenosis +/− other	16 (31.4%)
Logistic euroSCORE II (Median, interquartile range)	0.79 (0.72–1.16)

*eGFR* Estimated glomerular filtration rate

Peroperative data are shown in Table 2. All patients received a LIMA graft of which most were anastomosed to the LAD as in situ grafts. Twelve patients underwent total arterial revascularization with either the LIMA alone (two patients) or in combination with the RIMA and/or a RA.

**Table 2:** Operative details regarding 51 patients undergoing on-pump coronary artery bypass grafting

Time of surgery skin-to-skin (minutes) (Median (Interquartile range)	240 (195–275)
Aortic cross clamp (minutes) (Median (Interquartile range)	65 (48.5–80.0)
Time of cardiopulmonary bypass (minutes) (Median (Interquartile range)	112 (82.5–128.0)
Number of grafts per patient (Median (Interquartile range)	3 (2.5–4)
Total number of peripheral anastomoses.	155
End-to-side	124
Side-to-side	31
Number of patients who received	
LIMA	51
RIMA	8
SVG	39
Radial artery	15
Anastomoses from which ultrasonography was obtained in six planes (n; %).	
All patients	134/153 (88%)
After the initial 15 patients	100/108 (93%)

*LIMA* Left internal mammary artery, *RIMA* Right internal mammary artery, *SVG* Saphenous vein graft

ECUS was performed in 153 of the anastomoses. All ultrasound projections were obtained from 134 of these anastomoses (87%), and after the initial 15 patients all six projections were obtained in 100 of 108 scanned anastomoses (93%). The reasons why all images could not be obtained from a specific anastomosis were as follows: a lack of coagulated blood and approved ultrasound gel for obtaining optimal acoustic contact between the ultrasound probe and the anastomosis; a curved sequential graft with an anastomoses that could not be contained in the straight cavity of the Echoclip device; echo artefacts from a Titanium clip located in the roof of an anastomoses; and challenges in interpreting the images during the learning curve.

**Fig. 3:**
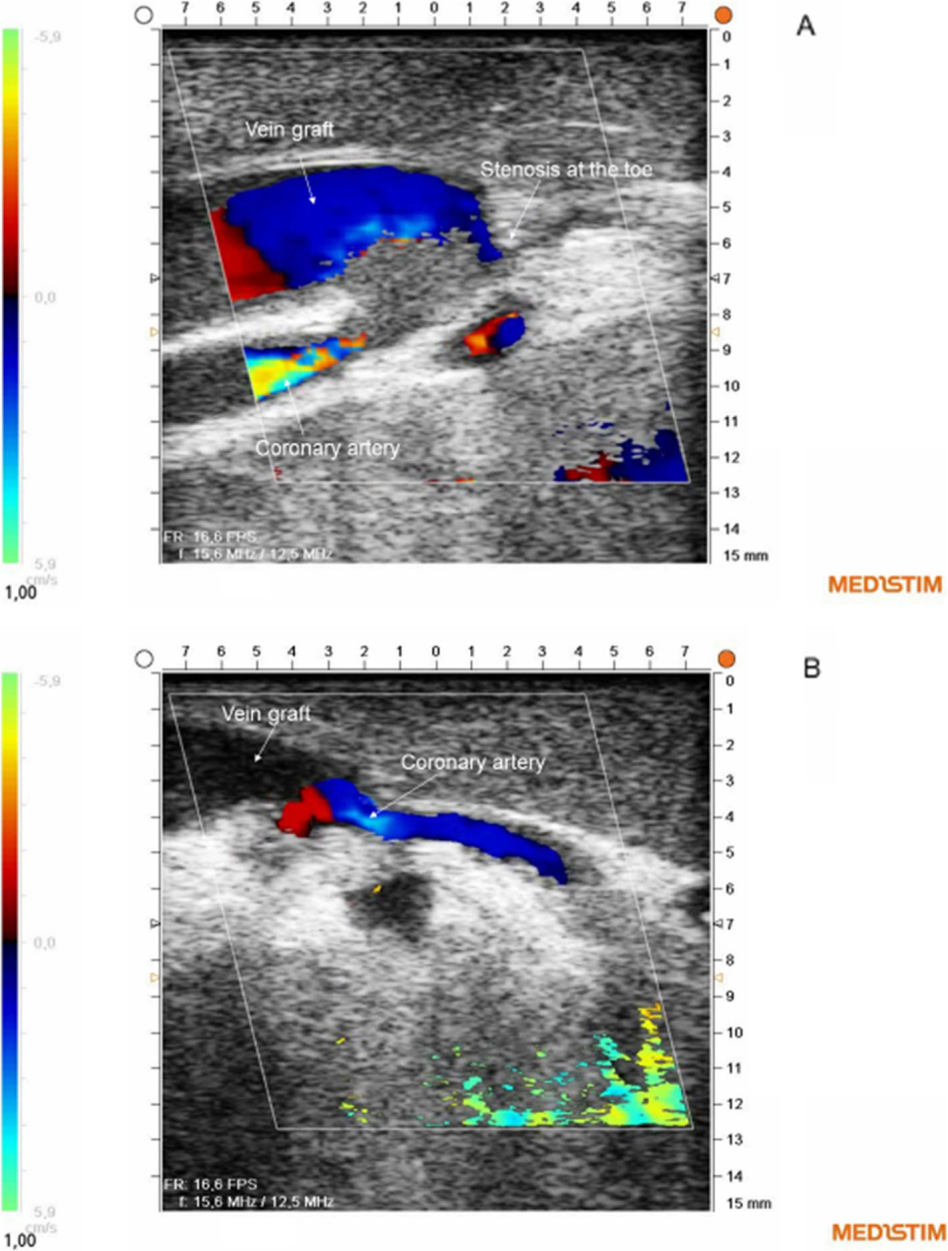
Doppler colour flow images showing a vein graft anastomosis with stenosis at the toe (**a**) and after revision of the anastomoses (**b**)

Combined use of TTFM and ECUS resulted in immediate revision of a peripheral anastomosis in five different patients: TTFM was not acceptable in the LIMA graft in two patients (Flow < 20 ml/min; PI: > 5), and in a third patient TTFM indicated a problem with a RIMA sequential side-to-side anastomosis to an intermediate coronary artery (Flow < 20 ml/min, PI: > 5). ECUS indicated a graft problem in two of these patients, and ECUS was not obtained from the side-to-side anastomosis in the third. During revision of the anastomoses a stenosis due to a suture was identified in two anastomoses, and no explanation was found in a LIMA graft. A spasm in the graft was suspected. Flow was increased in all anastomoses after revision. A fourth patient had an SVG anastomosed to a marginal coronary artery with no indication of a flow problem while infusing cardioplegia into the graft. However, ECUS showed antegrade obstruction at the toe of the anastomosis (Fig. 3). In a fifth patient, ECUS indicated an obstruction in a LIMA to the LAD anastomosis while the TTFM was acceptable (Flow: 26 ml/min; PI: 1.8¸ DF: 69%). Revision of these anastomoses showed an obstructing intimal flap at the toe in both anastomoses.

No patients suffered a perioperative myocardial infarction within 30 days, but one patient experienced recurrent angina and ventricular fibrillation in the ward on postoperative day four. After cardiopulmonary resuscitation, a coronary re-angiography showed low flow from the LIMA to the LAD and spasm with thrombus formation in the sequential RA graft distal to the first and a still-patent side-to-side anastomosis to a diagonal branch. The thrombus was causing occlusion of the anastomoses to the intermediate and the first obtuse marginal artery. The patient was brought to the operating room, and new anastomoses were constructed. The patient was discharged after 1 week following the re-operation without any physical sequelae. ECUS did not indicate any problems during the primary operation, and TTFM did not indicate any problems with the sequential graft flow either. However, TTFM was only performed proximally for the first anastomosis of the sequential graft and distally near the end-to-side anastomosis. Flow to the intermediate coronary artery could not be measured due to a titanium clip on the graft. ECUS was not performed during the re-operation. The patient had received calcium antagonists according to the postoperative routine.

A postoperative coronary re-angiography was performed in three patients in addition to the case mentioned above. One patient developed ST- elevations in the ECG 16 h postoperative, and an occlusion of the distal end-to-side anastomosis of a sequential radial artery to a small obtuse marginal was diagnosed. A re-angiogram was performed in another patient with the intention of performing percutaneous stenting, as no branches from the circumflex artery were identified during surgery. All anastomoses were patent in this patient, and no additional revascularisation could be performed due to small target vessels. The fourth patient was referred for percutaneous revascularisation of the right coronary artery as this vessel could not be re-vascularized during surgery. The angiography showed thrombosis of a vein graft to a small obtuse marginal artery, and no further attempts to re-vascularize this branch were pursued. The patient has no signs or symptoms of myocardial ischaemia.

One patient suffered a stroke. It turned out that this patient had bilateral stenoses of the internal carotid arteries. No patient experienced symptoms of TCI. New-onset postoperative atrial fibrillation was diagnosed in 18 patients (36%). Four patients underwent re-exploration for bleeding. Surgical bleeding was found in two patients. Five patients developed acute stage 1 kidney injury according to the KDIGO criteria [11], and none developed deep sternal wound infections. There was no 30-day or in-hospital mortality.

**Fig. 4:**
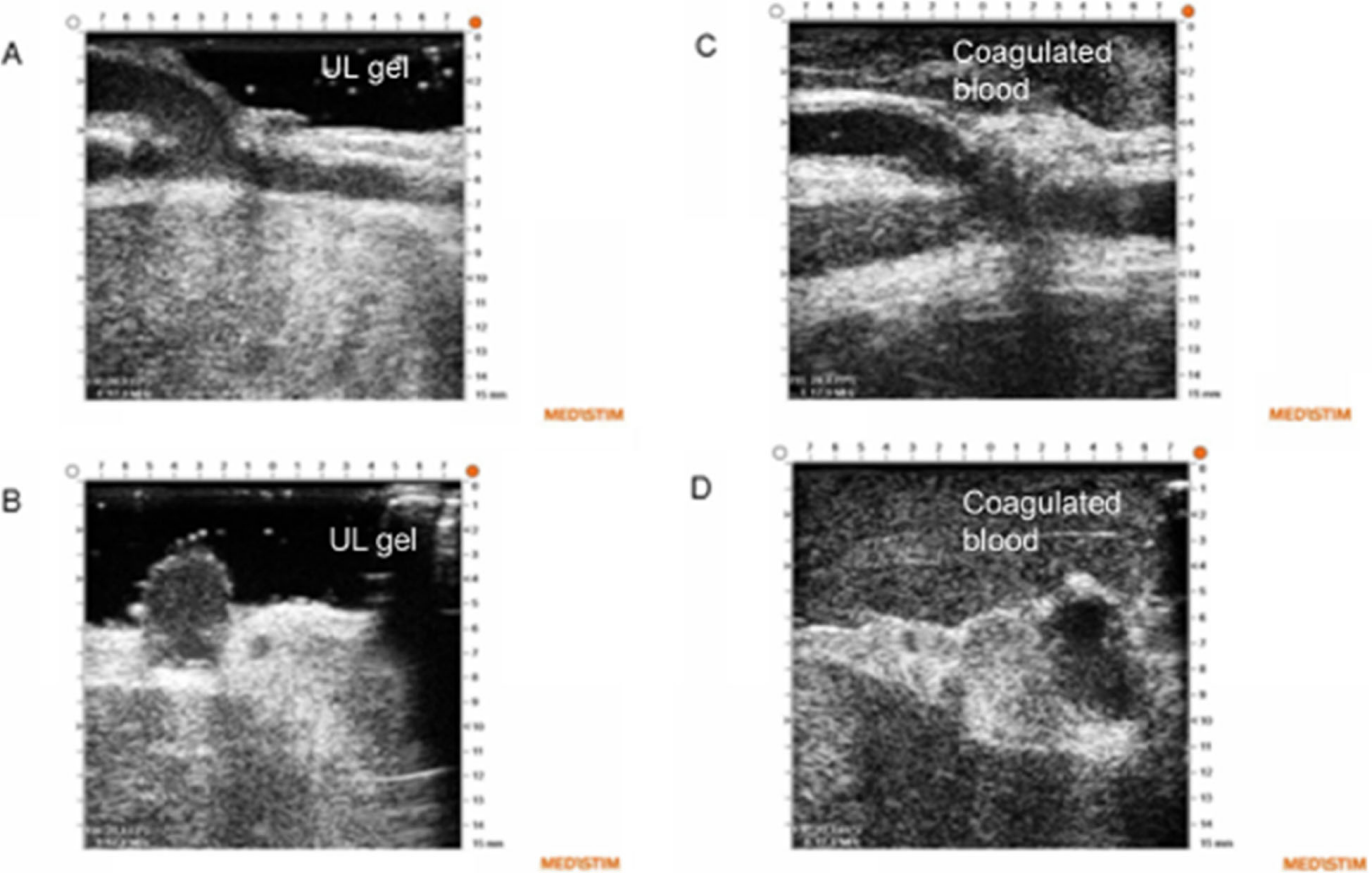
Epicardial ultrasonography of coronary anastomoses with either gel (A + B) or coagulated blood (C + D) as the acoustic coupling fluid

The blood clots that were used instead of ultrasound gel produced acoustic signals, and colour Doppler flow was often needed initially to aid in locating the anastomosis in the image at the VeriQ system. When this was realized, the time required for complete ECUS assessment of an anastomosis quickly decreased to 1–1.5 min per anastomosis. More disturbing echo signals on top of the anastomosis were observed when imaging was performed with the use of coagulated blood instead of ultrasound gel (Fig. 4).

## Discussion

In this prospective clinical feasibility study of 51 on-pump elective CABG patients with a total of 155 peripheral coronary bypass anastomoses, we have shown that intraoperative use of the Echoclip stabilizing device during ECUS successfully facilitates safe imaging in two planes of anastomoses located in all coronary territories. As a secondary result, we have shown that ECUS using the Echoclip device offers the surgeon the possibility of detecting and revising a graft failure that would otherwise not have been identified by the use of TTFM alone.

Early graft occlusions have been described to occur in 5–10% of the grafts in patients undergoing CABG [12]. Failures that may be detected during intraoperative graft assessment include twisting of the graft, anastomosis stenosis, a narrow toe of the anastomosis, kinked graft, etc. Ultimately, intraoperative ECUS with the use of a stabilizing device may limit the need for repeat revascularization and help reduce adverse events resulting from technical graft failures early after CABG. In the present study, the surgeons revised five (3.2%) peripheral anastomoses based on combined TTEM and ECUS due to technical failures. In two of these cases, TTFM did not indicate any graft problems. These graft failures may have resulted in signs and symptoms of graft failure by the end of surgery, but early graft occlusion may be silent [13]. Technical failures that are not detected during the operation may increase the risk of postoperative ischaemia, myocardial infarction or sudden death in the postoperative period.

In the present study, we arbitrarily decided to include 100 patients. However, after having included more than half of the intended number of patients, we realized that we did not obtain any further technical knowledge on how to use the Echoclip device in different coronary territories. Therefore, we decided to end the study. Furthermore, as we did not aim to predict early graft failure and clinical outcomes based on the results from this study, we found the number of included patients to be sufficient for a feasibility study.

There were no adverse events suspected to be associated with the use of the Echoclip device. One patient experienced a major cardiac event on postoperative day four. Manipulation of arterial grafts, which may happen during ECUS, may potentially have cause spasm in the grafts and caused occlusion. However, due to the time from ECUS to the major cardiac event, we find it reasonable to conclude that this was unrelated to the intraoperative ECUS procedure. The number of patients experiencing mild, acute kidney injury is in accordance with the incidence described in the literature following cardiac surgery. Early postoperative kidney injury may be diagnosed with an incidence between 8.9–39% following cardiac surgery depending on the type of surgery and diagnostic criteria [14, 15]; although there was a relatively high number of reoperations due to bleeding (8%), we have no reason to believe that this was related to the ECUS procedure.

Thus, clinical evidence from the present study supports the safety of the stabilizing device. To our knowledge, there is no published evidence in the English literature of adverse events in relation to ECUS in general.

We did not compare the effectiveness of performing ECUS with and without the Echoclip device. However, the use of a stabilizing device may be a more effective alternative compared with ECUS without the device because use of the device offers the surgeon the opportunity to keep the ultrasound transducer in air-free contact with the anastomosis without the risk of deforming the anastomosis while looking at the ultrasound images at the screen.

Sterile water-soluble ultrasound gel was used as the acoustic coupling fluid in the initial four patients. During surgery, the gel may be sucked from the pericardium into the heart-lung machine, and the gel may eventually end up in the patient’s circulation. However, there is no information in the literature on whether this may cause any harmful effects. Use of coagulated blood instead of gel has several limitations in addition to producing more noisy echo signals. First, blood clots are problematic to use, as the surgeon must remember to collect blood from the patient before heparinization. Second, clotted blood is a limited resource after heparinization, and third, blood clots are more slippery than gel. This makes it more difficult to keep the blood clot in place inside the Echoclip device compared with gel. In contrast with the blood clot, gel can be applied after the stabilizing device with the echo probe is positioned on top of an anastomosis. An ultrasound gel should be developed and approved for application in the pericardial space during cardiac surgery.

Surgeons must overcome a learning curve in relation to the use of ECUS of peripheral coronary anastomoses. Experienced surgeons have indicated that the basics can be learned in approximately 10–20 grafts [16, 17]. This is consistent with the learning curve experienced in the present study.

Interpreting ECUS images may be difficult. However, recent studies have proposed methods for automatic anastomosis segmentation, which may provide quantitative analysis of ECUS images to aid the interpretation in clinical practice in the future [18].

In addition to peroperative graft assessment, ECUS can also be used for surgical guidance, e.g., epi-aortic scanning in order to identify non-palpable soft arteriosclerotic plaques, detect coronary vessels embedded in the myocardium, and assess native coronary arteries for the optimal site for anastomoses [17]. This can be done without a stabilizing device.

In addition to a potential patient impact of ECUS with the stabilizing device, there may also be a potential system impact. The possibility of revising a graft failure before the patient leaves the operation room was observed, and this may be a key benefit to the healthcare system; however, we did not perform a health economic analysis. The Echoclip device is still not commercially available, but additional costs of the Echoclip technology will probably only have a minor impact on resources, especially if a MiraQ system is already available. Use of ECUS and the stabilizing device requires minimal training and has a short learning curve.

Some limitations of this study must be mentioned. First, although several surgeons participated in interpreting the images during surgery in order to identify the heel, middle and toe of the anastomoses in two planes, a majority of the ECUS procedures were performed by a single surgeon. Therefore, the results regarding the learning curve may not be generalizable. Second, patients were not included consecutively for practical reasons. Thus, there is risk of bias with regard to the inclusion of patients. After having included the initial 15 patients, we included patients based on availability of the surgeon who was most experienced with the use of the Echoclip device, but we have no reason to believe that patients were systematically included based on specific clinical and para-clinical data. Third, a coronary angiogram, which is considered to be the most accurate technique for graft assessment, was not performed per- or postoperative as a routine, and therefore we cannot evaluate the diagnostic accuracy of the ECUS procedures performed in this feasibility study. Fourth, we did not compare TTFM to the ECUS findings; however, as we did not aim to evaluate the combined use of TTFM and ECUS, we consider this a minor limitation. Fifth, the study was performed in a small centre with a relatively small number of patients. Furthermore, the study was limited to on-pump surgery.

Future studies on the Echoclip device will be conduct to perform blinded comparisons of UL images obtained from the same anastomosis with and without use of the device using algorithms for automatic estimation [18] of the anastomotic quality. Furthermore, the Echoclip device will be tested during off-pump surgery. An ultrasound gel to be approved for application in the pericardial space should be developed, and we also intend to obtain a CE marking (Conformité Européenne) for the Echoclip device as a symbol of free marketability in the European Economic Area.

## Conclusions

Peroperative use of a stabilizing device during ultrasonography of coronary artery bypass anastomoses during on-pump surgery facilitates imaging and provides surgeons with non-deformed longitudinal and transverse images of all parts of the anastomoses in all coronary territories. Peroperative ECUS in addition to flow measurements has the potential to increase the likelihood of detecting technical errors in constructed anastomoses.

## Data Availability

All data presented are available from the corresponding author upon reasonable request.

## Abbreviations

CABG: Coronary artery bypass grafting
DF%: Diastolic filling percentage
ECG: Electrocardiography
ECUS: Epicardial ultrasonography
LAD: Left anterior descending artery
LIMA: Left internal mammary artery
PI: Pulsatility index
RA: Radial artery
RIMA: Right internal mammary artery
SVG: Saphenous vein graft
TCI: Transitory cerebral ischaemia
TTFM: Transit-time flow measurement

## References

[1] Mack MJ (2008). Intraoperative coronary graft assessment. Curr Opin Cardiol.

[2] Ohmes LB, Di Franco A, Di Giammarco G, Rosati CM (2017). Techniques for intraoperative graft assessment in coronary artery bypass surgery. J Thorac Dis.

[3] Thuijs Daniel J F M, Bekker Margreet W A, Taggart David P, Kappetein A Pieter, Kieser Teresa M, Wendt Daniel, Di Giammarco Gabriele, Trachiotis Gregory D, Puskas John D, Head Stuart J (2019). Improving coronary artery bypass grafting: a systematic review and meta-analysis on the impact of adopting transit-time flow measurement. European Journal of Cardio-Thoracic Surgery.

[4] Hiratzka LF, McPherson DD, Brandt B, Lamberth WC (1987). The role of intraoperative high-frequency epicardial echocardiography during coronary artery revascularization. Circulation.

[5] Di Giammarco G, Canosa C, Foschi M, Rabozzi R (2014). Intraoperative graft verification in coronary surgery: increased diagnostic accuracy adding high-resolution epicardial ultrasonography to transit-time flow measurement. Eur J Cardiothorac Surg.

[6] Andreasen JJ, Nøhr D, Jørgensen AS (2019). A case report on epicardial ultrasonography of coronary anastomoses using a stabilizing device without the use of ultrasound gel. J Cardiothorac Surg.

[7] Budde RPJ, Bakker PFA, Gründeman PF, Borst C (2009). High-frequency epicardial ultrasound: review of a multipurpose intraoperative tool for coronary surgery. Surg Endosc.

[8] Jokinen JJ, Werkkala K, Vainikka T, Peräkylä T, Simpanen J, Ihlberg L (2011). Clinical value of intra-operative transit-time flow measurement for coronary artery bypass grafting: a prospective angiography-controlled study. Eur J Cardiothorac Surg.

[9] Kolh P, Windecker S, Alfonso F, Collet JP (2014). 2014 ESC/EACTS Guidelines on myocardial revascularization. Eur J Cardiothorac Surg.

[10] Thygesen K, Alpert JS, Jaffe AS, Chaitman BR (2018). Fourth universal definition of myocardial infarction. Circulation.

[11] Khwaja A (2012). KDIGO clinical practice guidelines for acute kidney injury. Nephron Clin Pract.

[12] Bassiri HA, Salari F, Noohi F, Motevali M (2010). Predictors of early graft patency following coronary artery bypass surgery. Cardiol J.

[13] Rings L, Zientara A, Dzemali O, Odavic D (2019). Early silent graft in off-pump coronary artery bypass grafting: a computed tomography analysis. Eur J Cardiothorac Surg.

[14] Mao H, Katz N, Ariyanon W, Blanca-Martos L (2013). Cardiac surgery-associated acute kidney injury. Cardiorenal Med.

[15] Hansen MK, Gammelager H, Jacobsen C-J, Hjortdal VE (2014). Acute kidney injury and long-term risk of cardiovascular events after cardiac surgery: a population-based cohort study. J Cardiothorac Vasc Anesth.

[16] Kieser TM (2017). Graft quality verification in coronary artery bypass graft surgery: how, when and why?. Curr Opin Cardiol.

[17] Kieser TM, Taggart DP (2018). The use of intraoperative graft assessment in guiding graft revision. Ann Cardiothorac Surg.

[18] Jørgensen AS, Schmidt SE, Staalsen NH, Østergaard LR (2016). An improved algorithm for coronary bypass anastomosis segmentation in epicardial ultrasound sequences. Ultrasound Med Biol.

